# Mitochondrial fitness and cancer risk

**DOI:** 10.1371/journal.pone.0273520

**Published:** 2022-10-12

**Authors:** Andrew V. Kossenkov, Andrew Milcarek, Faiyaz Notta, Gun-Ho Jang, Julie M. Wilson, Steven Gallinger, Daniel Cui Zhou, Li Ding, Jagadish C. Ghosh, Michela Perego, Annamaria Morotti, Marco Locatelli, Marie E. Robert, Valentina Vaira, Dario C. Altieri

**Affiliations:** 1 Center for Systems and Computational Biology, The Wistar Institute, Philadelphia, PA, United States of America; 2 Immunology, Microenvironment and Metastasis Program, The Wistar Institute, Philadelphia, PA, United States of America; 3 Ontario Institute for Cancer Research, Toronto, ON, Canada; 4 Wallace McCain Centre for Pancreatic Cancer, Princess Margaret Cancer Centre, University Health Network, Toronto, ON, Canada; 5 Lunenfeld Tanenbaum Research Institute, Mount Sinai Hospital, Toronto, ON, Canada; 6 Hepatobiliary/Pancreatic Surgical Oncology Program, University Health Network, Toronto, ON, Canada; 7 Department of Medicine, Oncology Division, Washington University in St. Louis, St. Louis, MO, United States of America; 8 Division of Pathology, Fondazione IRCCS Ca’ Granda Ospedale Maggiore Policlinico, Milan, Italy; 9 Department of Pathophysiology and Transplantation, University of Milan, Milan, Italy; 10 Division of Neurosurgery, Fondazione IRCCS Ca’ Granda Ospedale Maggiore Policlinico, Milan, Italy; 11 Department of Pathology, Yale University School of Medicine, New Haven, CT, United States of America; OUHSC: The University of Oklahoma Health Sciences Center, UNITED STATES

## Abstract

Changes in metabolism are a hallmark of cancer, but molecular signatures of altered bioenergetics to aid in clinical decision-making do not currently exist. We recently identified a group of human tumors with constitutively reduced expression of the mitochondrial structural protein, Mic60, also called mitofilin or inner membrane mitochondrial protein (IMMT). These *Mic60-low* tumors exhibit severe loss of mitochondrial fitness, paradoxically accompanied by increased metastatic propensity and upregulation of a unique transcriptome of Interferon (IFN) signaling and Senescence-Associated Secretory Phenotype (SASP). Here, we show that an optimized, 11-gene signature of *Mic60-low* tumors is differentially expressed in multiple malignancies, compared to normal tissues, and correlates with poor patient outcome. When analyzed in three independent patient cohorts of pancreatic ductal adenocarcinoma (PDAC), the *Mic60-low* gene signature was associated with aggressive disease variants, local inflammation, FOLFIRINOX failure and shortened survival, independently of age, gender, or stage. Therefore, the 11-gene *Mic60-low* signature may provide an easily accessible molecular tool to stratify patient risk in PDAC and potentially other malignancies.

## Introduction

Tumors invariably rewire their metabolism to promote cellular plasticity, adapt to ever-changing nutrient availability and acquire traits of aggressive disease, including metastatic competence [1]. Cancer metabolism has long been equated with the preferential utilization of glycolysis by tumor cells even when oxygen is present [2], the so-called Warburg effect [3]. However, it is now clear that mitochondria are also important players in tumor metabolism, as exploitation of oxidative bioenergetics [4, 5], redox balance [6], multiple cell survival mechanisms [7], and *retrograde* nuclear gene expression [8] influences primary and metastatic disease [9, 10].

Although mechanisms of mitochondrial reprogramming in cancer have recently come into better focus [11–13], the role of organelle *fitness* in this process has not been widely considered. In fact, the microenvironment of tumor growth is highly unfavorable to mitochondria [14], as erratic oxygen concentrations and oxidative radicals can compromise organelle integrity, deregulate multiple mitochondrial functions, and activate cell death [15]. How mitochondria cope with the loss of fitness has remained controversial [16] and the impact of subpar or damaged mitochondria on tumor traits is not understood.

Recently, we identified a subset of human tumors with constitutively reduced levels of Mic60 [17], a mitochondrial structural protein and essential component of the MICOS complex [18], compared to normal tissues. Despite acute loss of mitochondrial fitness, bioenergetics defects and oxidative damage, *Mic60-low* tumors remain viable, paradoxically become more metastatic, and upregulate a novel transcriptome comprising effectors of interferon (IFN) signaling and Senescence-Associated Secretory Phenotype (SASP) [17].

Here, we examined the impact of the *Mic60-low* transcriptome on cancer risk.

## Materials and methods

### Patient samples

Primary patient samples with histologically confirmed diagnosis of normal brain parenchyma (tumor-free surgical margins, N = 5), low grade gliomas (LGG, N = 4, oligodendroglioma, astrocytoma) and glioblastoma (GBM, N = 6) were examined for differential expression of the *Mic60-low* gene signature by qPCR. For the glioma series, fresh-frozen material was available from Fondazione IRCCS Ca’ Granda Hospital under Institutional Review Board protocol n. 275/2013 and written informed consent from all patients was obtained before surgery. Clinically-annotated patient samples with confirmed histologic diagnosis of PDAC (N = 5) were obtained from the archival database of the Department of Pathology at Yale New Haven Hospital upon approval from the Yale University Institutional Review Board and examined for Mic60 expression by immunohistochemistry (IHC). All methods were performed in accordance with the relevant guidelines and regulations using fully de-identified patient material.

### Cell culture experiments

Human PDAC cell lines PANC-1 and CAPAN-2 (American Type Culture Collection, Manassas, VA) were transfected with control non-targeting siRNA or Mic60-directed siRNA in the presence of 25 nM Lipofectamine RNAiMAX (Invitrogen) at a 1:1 ratio (vol siRNA 20 μM/vol Lipofectamine RNAiMAX), as described [17]. Transfected cells were validated for Mic60 knockdown by Western blotting of total cell extracts prepared in 150 mM NaCl, 1% Triton X-100, 0.5% sodium deoxycholate, 0.1% SDS, 50 mM Tris, pH 8.0 in the presence of EDTA-free Protease Inhibitor Cocktail (Roche) and Phosphatase Inhibitor Cocktail (Roche). In some experiments, transfected PDAC cells were analyzed for cell migration on 8 μm PET inserts or cell invasion across Matrigel-coated inserts, as described [17].

### mRNA expression

PANC-1 and CAPAN-2 cells transfected as indicated above were harvested and RNA was immediately extracted using Quick-RNA Microprep (Zymo Research) according to the manufacturer’s instructions. cDNA was prepared with High32 Capacity cDNA Reverse Transcription Kit with RNase Inhibitor (ThermoFisher Scientific) and the reverse-transcription reaction performed on a BioRad T100 Thermal Cycler. Quantitative PCR was performed with SYBR™ Select Master Mix (ThermoFisher) on ABI Quant Studio 5 machine (ThermoFisher).

### IHC

Four μm-thick sections from tissue blocks of human PDAC tissue samples were stained with a primary antibody to Mic60 (BD Biosciences) using Benchmark Ultra Roche Ventana Immunostainer (Roche Group, Tucson, AZ) and diaminobenzidine (DAB) as a chromogen. All slides were counterstained with hematoxylin.

### TCGA analysis

As discovery dataset, log2-transformed mRNA expression values were downloaded from 33 tumor samples in The Cancer Genome Atlas (TCGA) database of the UCSC Xena browser (Pan-Cancer Atlas Hub) [19]. Average expression values of the *Mic60-low* gene signature were examined in tumor sets with RNA-seq data with matching normal tissues. Multivariate Cox regression was used to determine the effect of age, gender, and stage as co-factors on outcome. Cox regression p values and hazard ratios (HR) between patients based on median Mic60 signature values were obtained.

### COMPASS cohort

The Comprehensive Molecular Characterization of Advanced Pancreatic Ductal Adenocarcinomas (PDAC) for Better Treatment Selection (COMPASS) trial is a prospective multi-institutional Canadian cohort study. Patient eligibility criteria for the study have been described previously [20] and require a radiologic or histologic diagnosis of locally advanced or metastatic PDAC suitable for combination chemotherapy, and consent to a fresh tumor biopsy prior to treatment start. In terms of eligibility criteria, biopsies can be taken from the primary lesion or any metastatic sites and patients must not have had prior treatment for advanced disease. Treatment decisions are at the discretion of their medical oncologist. Response to therapy is assessed using CT and measured using RECIST 1.1. Demographics and treatment details, including subsequent treatments, are prospectively collected using an electronic MEDIDATA database. The COMPASS trial has been approved by the Institutional Review Board at participating sites (University Health Network, Toronto, Ontario, Canada; MUHC Centre for Applied Ethics, Montreal, Quebec, Canada; and Queen’s University Health Sciences and Affiliated Teaching Hospitals Research Ethics Board, Kingston, Ontario, Canada); each patient provided written informed consent prior to study entry. Bioinformatics analysis of the 11-gene *Mic60-low* gene signature was carried out on transcriptomic data collected from all patients enrolled in the trial from December 2015 until May 2019 and follow-up censored on August 30, 2019 (N = 195).

### CPTAC cohort

The Clinical Proteomic Tumor Analysis Consortium (CPTAC) [21] comprises a total of 140 participants (74 males, 66 females between the age group of 31–85) collected by 11 different tissue source sites from 7 different countries. Clinical data were obtained from tissue source sites and aggregated by an internal Comprehensive Data Resource database that synchronizes with the CPTAC Data Coordinating Center. Demographics, histopathologic information, treatment and patient outcome information were collected and reviewed before deposition into the Proteomic Data Commons (PDC) and Genomic Data Commons (GDC). The cohort consists of 53% male (n = 74) and 47% female (n = 66). Age distributions [31–40 (2.9%), 41–50 (9.3%), 51–60 (16.4%), 61–70 (42.9%), 71–80 (25.7%), and 81–90 (2.9%)] and stage distributions [I (16.4%), II (42.9%), III (30.0%), and IV (6.4%)] of the patients reflect the general incidence of surgically resected PDAC.

### Statistical analysis

Two-tailed Student’s *t* test or Wilcoxon rank sum test was used for two-group comparative analyses. For multiple-group comparisons, ANOVA or Kruskal-Wallis test with post-hoc Bonferroni’s procedure were applied. All statistical analyses and graphing were performed using GraphPad software package (Prism 9.0) for Windows. A p value of <0.05 was considered statistically significant.

## Results and discussion

**Fig 1:**
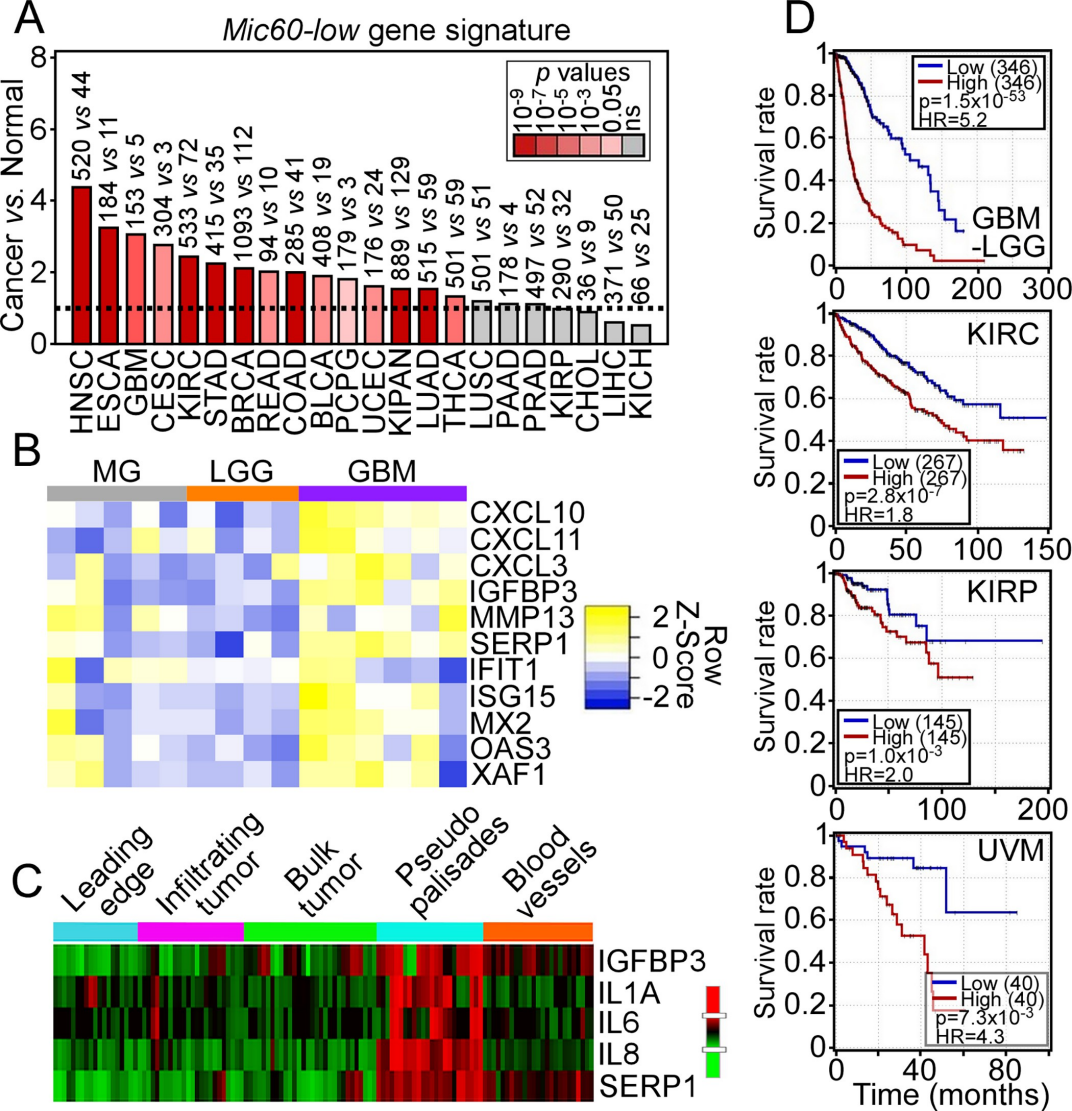
Differential expression of a *Mic60-low* gene signature in cancer. (A) TCGA tumor sets with RNA-seq data from matching normal tissues were examined for differential expression of the 11-gene *Mic60-low* gene signature in cancer *vs*. normal samples (ratio) by Wilcoxon rank-sum test. The number of tumor and normal tissue samples is indicated per each condition. Broken line, ratio of 1; red color scale, significance p value; ns, not significant. (B) Differential expression of the 11-gene *Mic60-low* gene signature in patient-derived tumor-free normal brain margins (MG), LGG or GBM by qPCR. The expression intensity of each gene is visualized in a heatmap. (C) The indicated representative genes in the *Mic60-low* gene signature were analyzed for spatial distribution in patient-derived GBM samples using the Ivy Glioblastoma Atlas Project dataset. The various GBM intratumoral compartments are indicated. (D) Kaplan-Meier survival curves of high *vs*. low expression of the *Mic60-low* gene signature in TCGA datasets of GBM-LGG, kidney renal clear cell carcinoma (KIRC), kidney renal papillary cell carcinoma (KIRP) and uveal melanoma (UVM). The number of patients per condition, p value and hazard ratio (HR) are indicated.

Recent studies have shown that tumors with reduced expression of the mitochondrial structural protein, Mic60 upregulate a unique transcriptome comprising regulators of IFN signaling and SASP [17]. In a first set of experiments, we narrowed the full, 52-gene *Mic60-low* transcriptome [17] to 11 genes representative of IFN response (*IFN Short*: IFIT1, ISG15, MX2, OAS3, XAF1) and SASP signaling (*SASP Short*: CXCL10, CXCL11, CXCL3, MMP13, IGFBP3, SERPINE1) based on >5-fold upregulation and role in cancer. In analysis of TCGA datasets, the 11-gene *Mic60-low* gene signature was differentially expressed in multiple, genetically unrelated tumor types (p<0.05), compared to the corresponding normal tissues (Fig 1A). The highly heterogeneous distribution of Mic60 in PDAC (see below) may explain why expression of the *Mic60-low* gene signature did not reach statistical significance in this tumor type, compared to normal tissue (see below). In validation studies using GBM as a tumor model (Fig 1A), the 11-gene *Mic60-low* gene signature was highly expressed in primary, patient-derived GBM tissue samples, compared to LGG and normal brain parenchyma, i.e. tumor-free margins (MG) (Fig 1B). Examination of the Ivy Glioblastoma Atlas Project dataset (https://glioblastoma.alleninstitute.org) revealed that representative genes in the *Mic60-low* gene signature, SERPINE1, IL8, IL6, IL1α and IGFBP3 spatially segregated within garland-like hypoxic hypercellular structures known as GBM pseudopalisades and associated with high invasiveness (Fig 1C). Consistent with more aggressive phenotype, high expression of the *Mic60-low* gene signature was associated with dramatically reduced overall patient survival in the GBM-LGG dataset of TCGA (p = 1.5x10^-53^, HR = 5.2) (Fig 1D). Similar results were observed in TCGA datasets of other tumor types, where increased expression of the *Mic60-low* gene signature was associated with shorted patient survival in kidney cancer (both KIRC and KIRP), uveal melanoma (UVM) (Fig 1D), testicular germ cell tumors (HR>4, p = 0.0074, N = 139), and thymomas (HR = 3.1, p = 0.036, N = 119).

**Fig 2:**
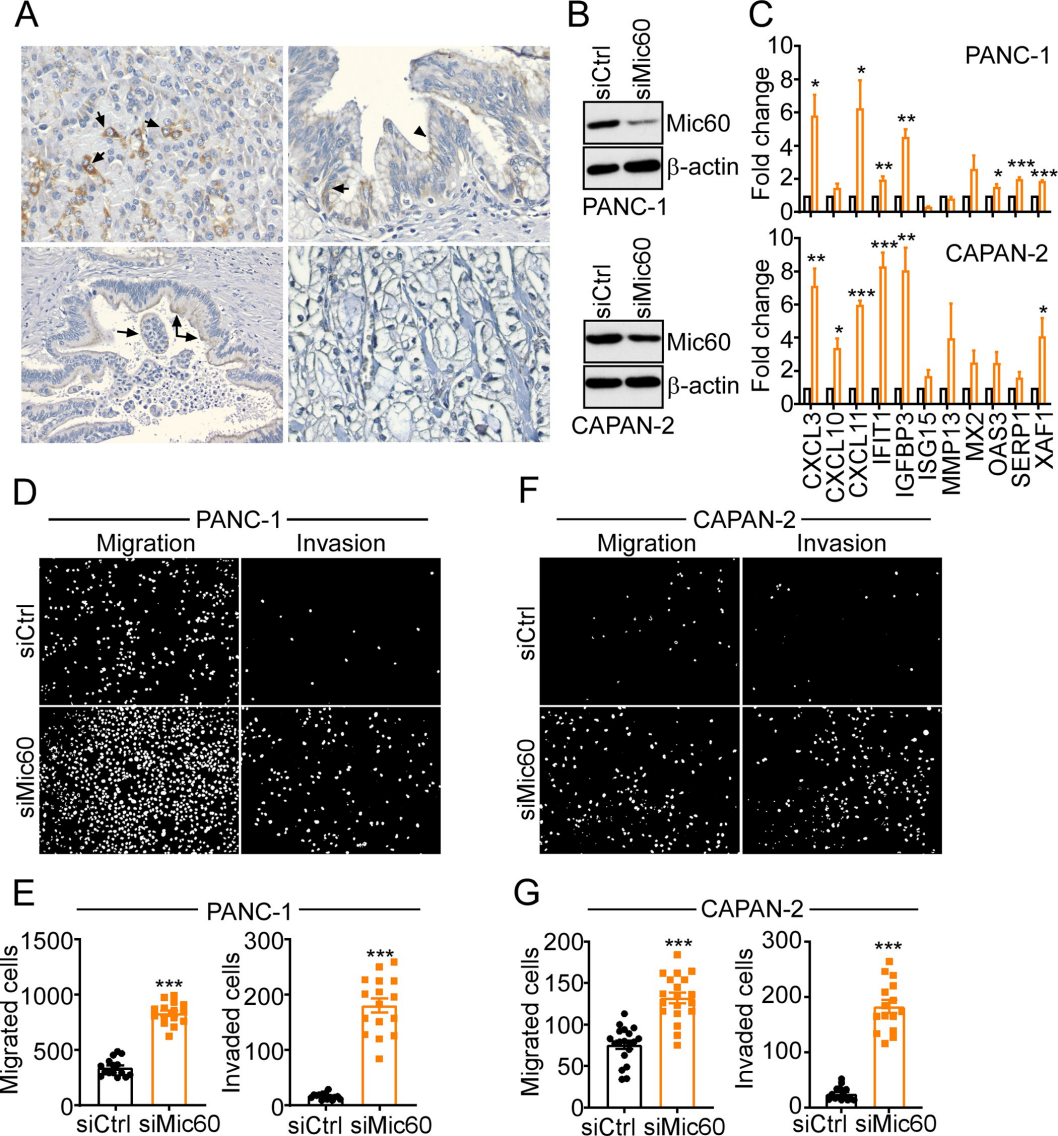
Characterization of Mic60 in pancreatic cancer. (A) Heterogeneous expression of Mic60 in PDAC patients by IHC. *Top Left*, arrows, perinuclear and cytoplasmic expression in normal pancreatic acini (x400). *Top Right*, perinuclear (arrow) and apical (arrowhead) cytoplasmic expression in high-grade pancreatic intraepithelial neoplasia (x400). *Bottom Left*, transition between Mic60-positive well differentiated tumor (double arrows) and Mic60-negative high-grade basaloid regions (single arrow) within the same tumor gland (x400). *Bottom right*, absent stain in high-grade basaloid PDAC (x400). A histoscore of Mic60 staining intensity was as follows: 0, absent; 1, low; 2, medium; 3, high. (B) PDAC cell lines PANC-1 (*top*) or CAPAN-2 (*bottom*) were transfected with control non-targeting siRNA (siCtrl) or Mic60-directed siRNA (siMic60) and analyzed by Western blotting. (C) The conditions are as in (B) and transfected cell lines were analyzed for differential expression of the indicated genes in the *Mic60-low* gene signature by qPCR. Mean±SEM (n = 3). *, p = 0.01–0.04; **, p = 0.001–0.004; ***, p<0.0001. (D-G) The conditions are as in (B) and transfected PANC-1 (D-E) or CAPAN-2 (F-G) cells were analyzed for cell motility and representative images of DAPI-stained nuclei of migrated or invaded cells were visualized by fluorescence microscopy (D-F) and quantified (E-G). Mean±SEM (n = 3). ***, p<0.0001.

Next, we focused on PDAC as a second tumor model to evaluate the Mic60 pathway in cancer. Consistent with recent observations [17], analysis of patient-derived tissue samples by immunohistochemistry demonstrated that Mic60 expression was highly heterogeneous in PDAC, with moderate expression in pancreatic intraepithelial neoplasia and well-differentiated PDAC to undetectable levels in high-grade, basaloid PDAC (Fig 2A).

To mimic the effect of reduced Mic60 levels in PDAC, we next silenced endogenous Mic60 expression in PDAC cell lines, PANC-1 and CAPAN-2 by siRNA (Fig 2B). Consistent with previous observations [17], Mic60 silencing was associated with increased expression of the *Mic60-low* gene signature in PDAC cell lines, albeit with cell type-specific differences (Fig 2C). Functionally, Mic60 silencing was associated with dramatically increased PANC-1 (Fig 2D and 2E) and CAPAN-2 (Fig 2F and 2G) cell migration and invasion.

**Fig 3:**
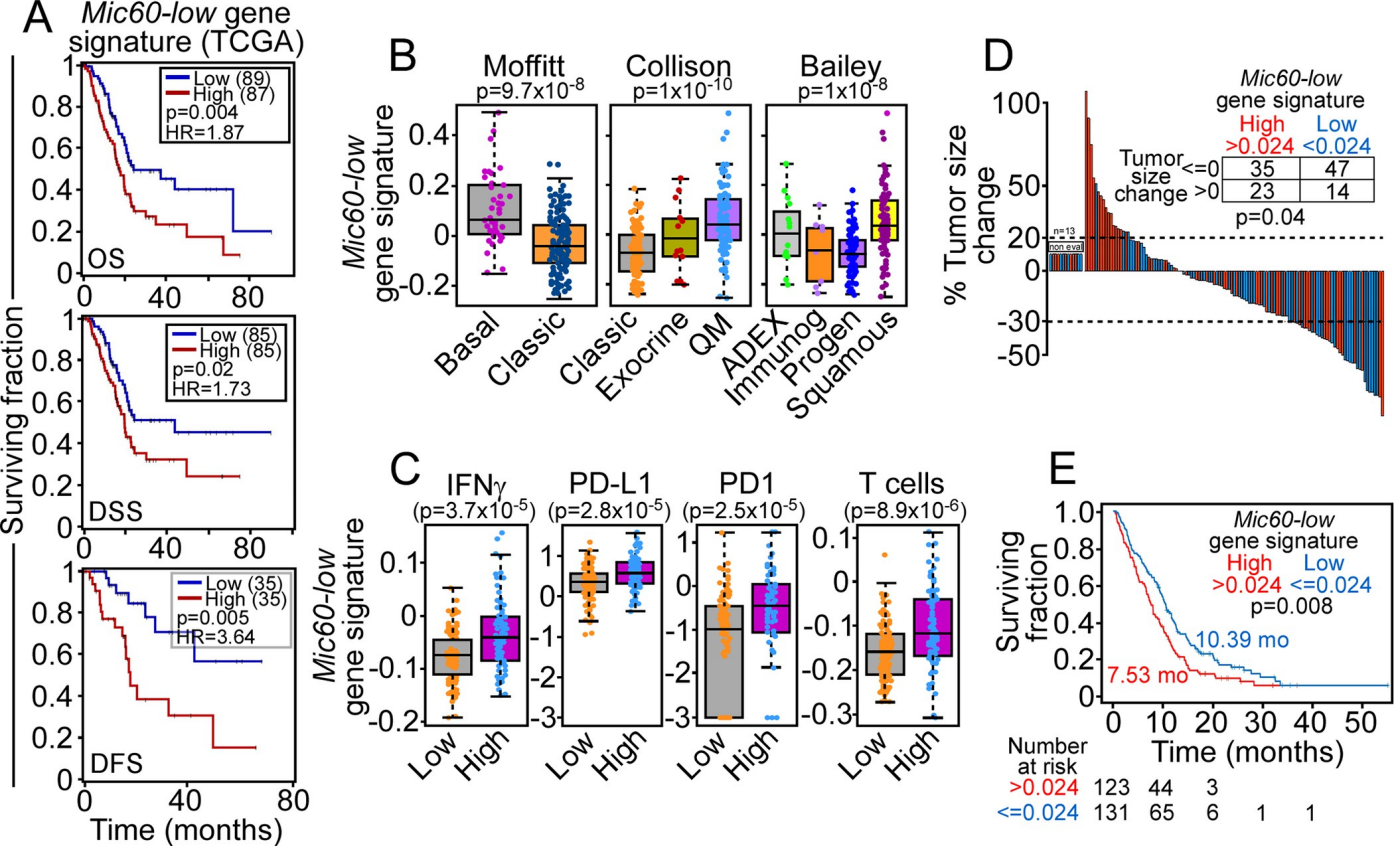
*Mic60-low* gene signature and PDAC risk. (A) Kaplan-Meier plots of PDAC overall survival (OS), disease-specific survival (DSS) and disease-free status (DFS) determined by univariate Cox regression tests in the TCGA datasets (N = 32). (B) Expression of the 11-gene *Mic60-low* gene signature in PDAC molecular subtypes by Wilcoxon rank sum test (Moffitt) and Kruskal Wallis test (Collison, Bailey). QM, quasimesenchymal; ADEX, aberrantly differentiated endocrine exocrine; Immunogen, immunogenic; Progen, pancreatic progenitor. (C) Modulation of inflammation-associated markers, IFNγ, PD-L1, PD1 and T cells all by Wilcoxon test. (D) Waterfall plot of tumor size changes in PDAC patients treated with FOLFIRINOX by Wilcoxon test. non-eval, non-evaluable. (E) Kaplan-Meier survival curve of PDAC overall survival (95% CI: 0.533–0.9118). Analyses were carried out by single-sample Gene Set Enrichment Analysis (ssGSEA) with *Mic60-low* gene signature high (>0.024) or low (< = 0.024) and Wilcoxon test.

Based on these data, we next assessed the impact of the *Mic60-low* gene signature on PDAC risk. First, expression of the *Mic60-low* gene signature in the TCGA dataset of PDAC was associated with shortened overall survival (HR = 1.87, p = 0.004, N = 176), disease-specific survival (HR = 1.73, p = 0.02, N = 170) and disease-free status (HR = 3.6, p = 0.005, N = 70), independently of age, gender, or stage (Fig 3A). Next, we examined RNA-Seq data from patients (N = 195) enrolled in the COMPASS trial [20]. In this patient cohort [20], increased expression of the *Mic60-low* gene signature was associated with aggressive molecular variants of basal, quasi-mesenchymal and squamous PDAC (Fig 3B), inflammation-associated expression of IFNγ (p = 4x10^-5^), PD1 (p = 3x10^-5^), PD-L1 (p = 3x10^-5^) and T cells (p = 9x10^-6^) (Fig 3C), FOLFIRINOX failure (Fig 3D) and shorter overall survival (Fig 3E). Conversely, the *Mic60-low* gene signature did not correlate with hypoxia status (p = 0.7, Wilcoxon rank sum test), cell cycle progression (p = 0.46, Wilcoxon rank sum test) [22] or four genomic subtypes of PDAC [23], including stable, locally rearranged, scattered and unstable (p = 0.39, Kruskal-Wallis test).

As a third dataset, we examined the expression of the *Mic60-low* gene signature in the CPTAC dataset [21]. In this cohort, higher values of the *Mic60-low* gene signature independently correlated with differential expression in PDAC *vs*. normal tissue (p = 0.006) and basal *vs*. classical molecular tumor variants (p = 0.005).

To the best of our knowledge, this is the first gene signature of mitochondrial reprogramming in cancer linked to aggressive disease subtypes, treatment failure and abbreviated patient survival. Despite progress in molecular [22] and genomic [23] profiling, the role of mitochondrial reprogramming in PDAC is only beginning to emerge [24] and metabolic biomarkers for detection and treatment in these settings remain urgently needed [25]. Mic60 is an essential structural protein in mitochondria and its reduced expression triggers acute organelle dysfunction, loss of bioenergetics and oxidative stress [17]. The basis for the reduced and often undetectable levels of Mic60 in many human tumors remains to be elucidated. However, we speculate that the unique transcriptome of SASP and IFN signaling upregulated in these *Mic60-low* tumors represents an adaptive response to loss of mitochondrial fitness that confers increased metastatic ability [17]. Accordingly, SASP signaling has been linked to increased tumor cell invasion and metastasis [26], whereas chronic activation of a type I IFN response enhances pro-tumorigenic inflammation [27] and local immunosuppression [28].

## Conclusions

Based on the findings presented here, the *Mic60-low* gene signature may provide an easily accessible, point-of-service molecular tool to stratify patient risk in PDAC and potentially other malignancies, including GBM. A potential limitation of our study is the undetermined clinical characteristics of PDAC and GBM patients expressing the *Mic60-low* gene signature.

## Supporting information

S1 File (DOCX)

S1 Raw images (PDF)
