# Supplementary material for: Mitochondrial fitness and cancer risk
Source: PLoS One. 2022 Oct 12;17(10):e0273520. doi: 10.1371/journal.pone.0273520 (PMC9555630; doi:10.1371/journal.pone.0273520)
Supplement: S1 File — (DOCX) [file pone.0273520.s001.docx]

**MITOCHONDRIAL FITNESS AND CANCER RISK**

Andrew V. Kossenkov, Andrew Milcarek, Faiyaz Notta, Gun-Ho Jang, Julie M. Wilson, Steven Gallinger, Daniel Cui Zhou, Li Ding, Jagadish C. Ghosh, Michela Perego, Annamaria Morotti, Marco Locatelli, Marie E. Robert, Valentina Vaira, and Dario C. Altieri

**SUPPLEMENTARY DATASET FILE**

**ORIGINAL IMAGES**

**Fig 2B** - Original uncut blots


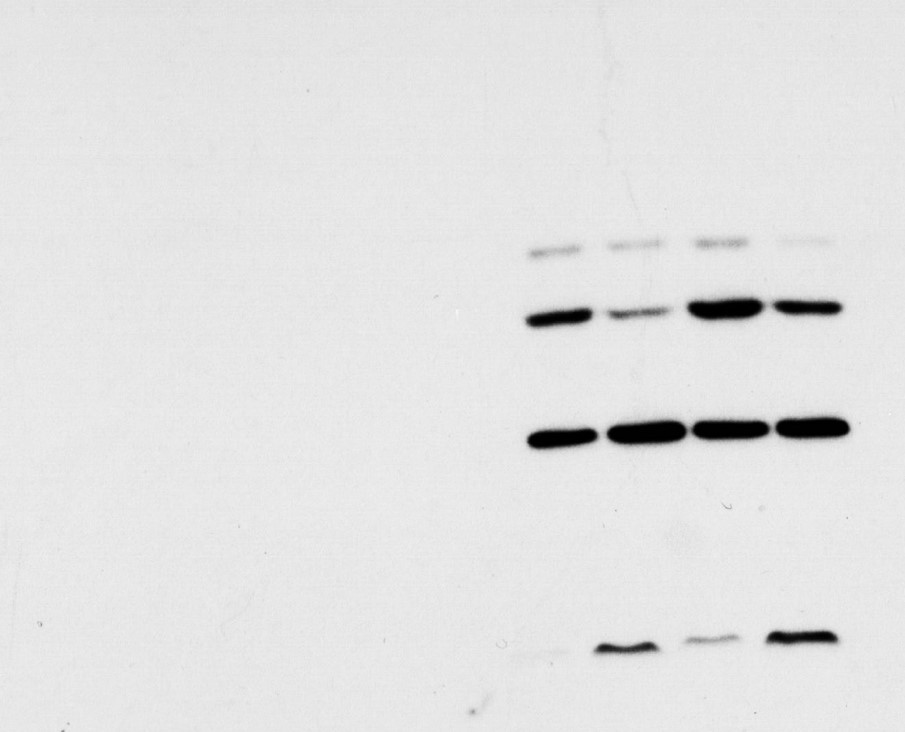


**MATERIAL AVAILABILITY**

The RNA-Seq dataset contained in this manuscript has been submitted with GEO accession GSE197465: <https://www.ncbi.nlm.nih.gov/geo/query/acc.cgi?acc=GSE197465>

**DATA ANALYSIS**

Data are presented as mean±SEM of at least three independent biological replicates. Two-tailed Student’s *t* test or Wilcoxon rank sum test was used for two-group comparative analyses. For multiple-group comparisons, ANOVA or Kruskal-Wallis test with post-hoc Bonferroni’s procedure were applied. All statistical analyses and graphing were performed using GraphPad software package (Prism 9.0) for Windows. A p value of ˂0.05 was considered statistically significant.
