# Supplementary figures and images for: Mitochondrial fitness and cancer risk
Source: PLoS One. 2022 Oct 12;17(10):e0273520. doi: 10.1371/journal.pone.0273520 (PMC9555630; doi:10.1371/journal.pone.0273520)

Fig. 2B -Uncut blot

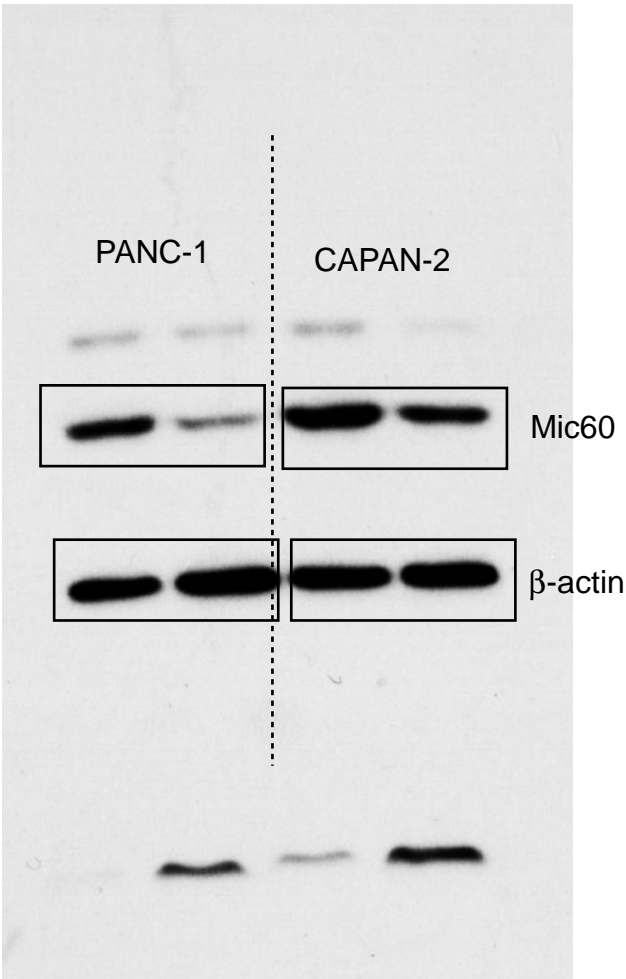

Supplement: S1 Raw images — (PDF) [file pone.0273520.s002.pdf]
